# Determinants of Travel Participation and Experiences of Wheelchair Users Traveling to the Bodrum Region: A Qualitative Study

**DOI:** 10.3390/ijerph18052218

**Published:** 2021-02-24

**Authors:** Ezgi Özcan, Zehra Güçhan Topcu, Hüseyin Arasli

**Affiliations:** 1 Faculty of Tourism, Eastern Mediterranean University, 99628 Famagusta, Cyprus; ezgiozcan0001@gmail.com; 2 Department of Physiotherapy and Rehabilitation, Faculty of Health Sciences, Eastern Mediterranean University, 99628 Famagusta, Cyprus; 3 Norwegian School of Hotel Management, Faculty of Social Sciences, The University of Stavanger, 4036 Stavanger, Norway; araslih@gmail.com

**Keywords:** wheelchair users, participation to travel, disabled, accessible tourism, holiday determinants, assistantship, emotional touch

## Abstract

Although the number of people with disabilities and types of disability increases day by day, a sufficient point has not been reached regarding accessible tourism. The participation rate of people with disabilities (PWDS) in tourism activities is low, and there is a big gap in the travel and accommodation sector in this regard. Studies of previous scholars have concluded that the accessible tourism market is a significant and profitable area, but determinants of participation to travel and process of travel, such as wheelchair user expectations, are consistently ignored by the tourism industry. The main purpose of this study is to determine the determinants of travel by examining the motivations, expectations, processes and experiences of PWDS using wheelchairs to participate in tourism. Research was performed in Turkey’s Bodrum district; 25 wheelchair users were included in the study. The keywords that emerged in the theoretical framework in light of the answers given to 39 open-ended questions online were coded in the Nvivo program. The results showed that wheelchair users intended to go on vacation but were less motivated to participate due to the lack of travel conditions. Wheelchair travelers argued that a companion was required for an enjoyable holiday that could meet their needs. In addition, the results revealed that the types of wheelchairs used by disabled passengers differ. The disabled stated that the wheelchairs they use in daily life are not suitable for use on the beach, sand or water. Despite the economic, social and technological change opportunities, basic tourism service expectations of PWDS are not met. This research project is a comprehensive study that makes determinations in terms of examining the social status of disabled people in terms of social sciences, examining the place and importance of disabled tourists in the market and eliminating the deficiencies of facilities serving in tourism.

## 1. Introduction

Tourism is a profitable and expanding sustainable market but also a social imperative that should include equal access to hospitality, products or services. Considering the increasing disabled population and its place in the market, the goal of accessible tourism is a research subject for the tourism and travel sector. However, the biggest consumption of our age, the internet and technology, offers various options for consumers to buy products and services from suppliers. Moreover, the importance of strategic tourism marketing planning and sustainability issues is increasing day by day for the tourism sector [1,2,3].

According to the data of the World Health Organization (WHO), it is conjectured that more than one billion people, in other words, 15% of the world population, live with various levels of disability. With the increase in disability types and population, we aim to examine the social situation of disabled people in developed countries, to increase their quality of life and to improve their accessibility (WHO, 2019). Disability is defined as the loss of physical, mental, emotional and social abilities as a result of organ defects or absence that causes loss of function due to congenital, accident or a prolonged illness. People with disabilities (PWDS) are defined as people who have a disability or a variety of disabilities, such as people with hearing loss or people with spinal cord injury. When the concept, definition and type of disability are correctly defined, numerous topics on health and social policy can be addressed, and appropriate studies can be contrived to evaluate what interventions can be made to advance the lives and well-being of all PWDS [4].

In Europe and many developed countries, sanctions protecting the rights and freedoms of the disabled have been imposed, attempts have been made to overcome access problems, and an alternative market has been created in tourism for the participation of disabled individuals in tourism activities with social policies, legal measures and disabled-friendly facilities [5]. The need to see disabled people as a part of society, make the necessary arrangements to enable them to live like other people in society and encourage them to increase their participation in tourism activities has been investigated [6]. The researchers emphasized the opportunities and obstacles in their studies on the demand of the disabled to participate in tourism and stated that “as the society develops, tourism becomes a necessity rather than a social desire” [7].

Considering the importance of disabled people’s social life and their place in tourism, it is seen that research and participation in tourism activities are limited [8]. The participation of disabled individuals in touristic activities like other members of society reduces their social exclusion and enables social peace to be formed. Although PWDS are very enthusiastic to participate in touristic activities, they cannot participate in these activities adequately due to their negative experiences on holiday. Structural and legal regulations are required in touristic regions for their participation in these activities. In addition, social conditions, such as family, profession, income level and treatment conditions of disabled tourists, affect their travel intentions. However, among the most important details are the suitability of the place of accommodation, transportation and access facilities and the ability of the service to be provided at the destination to meet the needs of the disabled individual [9]. According to Premović and colleagues [10], the contribution of the development of the tourist market to socio-economic and cultural development, and the effects of tourism on the world, should be highlighted.

Ultimately, children with disabilities and their families face many restrictions in terms of access, accommodation and transportation while traveling for tourism purposes, and this affects their final destination choices. Considering that PWDS go on vacation with at least one companion or their family, this makes them an important target audience in the tourism market [11].

The types of disability are numerous, as mentioned above, so it is important to focus on one type in studies. When we search the limited information on accessible tourism in the literature, there is a gap in the area of people who need a wheelchair for ambulation. Considering the limitations which they may face in touristic areas, environmental factors will probably have a more important role, but there is no adequate research showing the experiences of these people. Thus, the purpose of this research is to investigate the travel experiences of wheelchair users (WCu) in depth, observe details and perceptions, investigate the barriers they face in tourism, determine their expectations and the determinants of their participation in tourism and to shed light on new studies for development in terms of accessible tourism.

This aim can be stated in the following research questions:

1. What are the expectations of wheelchair users from the accessible tourism industry?
2. What are the main barriers and travel determinants that WCu face in terms of participation in tourism?
3. How do internal motivation and other environmental factors affect the motivation of PWDS to participate in travel?

This study examines the effects that determine the motivation of disabled people to participate in tourism and fully evaluates the participation processes of individuals who use wheelchairs for ambulation. While examining their experience in travel processes in detail, they understood their expectations from the tourism and travel industry and determined the determinants of their motivation to participate in travel. In addition, when examining the process and resulting relationship of a person using a wheelchair, their experience, determinants and expectations were fully addressed.

## 2. Background and Literature Review

### 2.1. Accessible Tourism and Accessibility

Today, with the increase in the number of disabled tourists and the enhancement in the types of accessible tourism, tourism participation movements and travel processes of disabled tourists in the tourism and travel sector are subject to examination and research.

Furthermore, increasing the participation of disabled people in tourism, meeting their special needs and expectations, ensuring that they benefit from all products or services, listening to their complaints and offering solutions are not only a sectoral duty but also a social responsibility. It is well known that PWDS have fewer opportunities and a poorer quality of life than those without disabilities, and they also have too many access problems.

Tourism is a complicated system in which accessibility, with heterogeneous qualities, is an important supplementary that interacts with the customers’ needs chain from the travel planning to the transit stage and the destination [12]. As social equity is regarded as a valuable goal, providing an environment that facilitates travel for people with various disabilities, both at the airport terminal and on board the aircraft, is becoming increasingly important for airlines and airport officials [13].

The recent literature on accessibility is based on sustainability and inclusivity, which will be the future goal and emphasizes the importance of the bridge between industry and society [14]. The most important obstacles to the participation of disabled people in tourism activities are economic, physical and transportation conditions. It has been proven that all kinds of tourism activities within the scope of accessible tourism can satisfy disabled people if these conditions are met [15].

Another study, which examined the limitations of the involvement of disabled people in travel and accessible tourism and focused on the tourism requirements of the disabled stated that there are studies that investigate the motivation, demand, choice, decision-making, experience and travel barriers around the tourism characteristics of disabled tourists [16]. Accessible tourism is a concept that goes beyond responding to the growing demand of the accommodation sector, and for an egalitarian society against discrimination, marginalization approaches towards disabled customers must be stopped [17]. Recent research has focused on the obstacles that affect the ability of PWDS to travel [18]. The increasing disabled population has become a key factor for the integration and competitiveness of concepts such as transportation, access and destinations in the tourism environment within global tourism [19].

Tourism is defined as an important indicator of quality of life. Therefore, the participation of a disabled person in tourism and their ability to benefit from services are important in terms of psychological health and travel needs. The results of the interviews with WCu show that the tourism and travel sector cannot meet the expectations regarding accessible tourism.

Considering the constantly increasing number and diversity of disabled individuals, it is observed that their place and importance in tourism should be increased by the fact that tourism provides an infrastructure for development and change, and its economic, social and cultural effects that determine living standards, tourism potential and the market for the disabled are of great importance in the travel and hospitality sector [7,20,21].

### 2.2. Accessible Tourism in Turkey

According to the Turkey Statistical Institute (TSI) (2019), there are 5 million physically disabled people out of 8 million disabled people in Turkey. The institute presents that the proportion of working disabled men is 35.4 percent, while the proportion of working disabled women is only 12.5 percent. The TSI also states that the biggest problem regarding the participation of disabled people in business and social life is accessibility: 42.8 percent of the physically disabled people are men and 57.2 percent are women.

Both a rapid increase in the disabled population in the world and in Turkey’s tourism sector, and that providers are monitoring the potential of the market segment, active strategies to maintain user-friendly services and barrier-free foundation applications, has implemented improvements and policies [22]. A disabled-friendly tourism facility should be an enterprise that is accessible, customized, developed, equipped and capable of meeting the needs and expectations of the disabled individual, and that offers complementary services [23].

The problems of disabled tourists during tourism activities with different types of obstacles in Turkey were identified to determine the demand characteristics. How much of the disabled population in Turkey has not been established to be involved in tourism activities? The limited economic conditions and inaccessible environmental conditions for disabled people can prevent their participation in tourism activities. To date, there is not a large amount of research conducted with regard to disability tourism in Turkey [24].

Turkey’s tourism sector demands an improvement in the quality of services offered to the disabled market, as well as the identification of difficulties experienced in this field, and in order to help domestic and foreign disabled guests to travel to our country, within TÜRSAB, 15 June 2006, the “Accessible Tourism Committee” was formed.

Various studies on accessible tourism are available in the literature, but little research has been conducted on the impact of disabled tourists on tourism. Turkey’s ideal travel agencies in the future will offer suggestions on the work that needs to be carried out and emphasize the importance of the place of disabled tourists in the industry [25].

In addition, there are almost no facilities where disabled people, elderly people and their families can have a holiday together with other people. However, making architectural arrangements is not sufficient for the participation of disabled people and their families in life and tourism. In order to change mentalities, public officials, private sector employees and the whole society should be given training on understanding the disabled, communicating without marginalizing and correct behavior.

### 2.3. Tourism in Bodrum

Turkey’s Aegean coast, located in the province of Muğla in Bodrum, Turkey, is among the world’s most popular resort locations. Bodrum is among Turkey’s most popular holiday destinations, and offers holidaymakers an unforgettable holiday experience, with its magnificent sea, natural beauties, pine forests, natural parks, bays and beaches, luxury resorts, hotels and resorts, restaurants, nightclubs and shopping opportunities.

The oldest settlement, Halicarnassus, was founded in Bodrum city, with its unique architecture and historic whitewashed houses decorated with bougainvillea; seeing the world from Turkey’s Aegean is the most important part of a holiday in this paradise. Along the 650 km coastline of the Bodrum peninsula, Bodrum, Bitez, Ortakent, Turgutreis, Gümüşlük, Kadıkalesi, Yalıkavak, Gündoğan, Türkbükü, Aspat, Karaincir, Akyarlar and many more settlements and beaches in Turkey are among the most exclusive and popular holiday resorts.

Bodrum in summer is among Turkey’s most popular holiday destinations not only for locals but also for foreign tourists. It is a popular holiday city with a rich content that offers a wide variety of environments where you can relax or mingle with the crowd, and it also provides opportunities such as airport, car rental, hotel variety, house rental, city transportation, attractions, rich cuisine, nightlife, shopping, festivals and concerts.

Famous for its local dishes and rich cuisine appealing to tourists, the city of Bodrum is also preferred by travelers for its unique flavors. Cultural experience, social interaction, sensory seeking and sensory attractiveness factors were determined in the conducted study to determine the motivations of international visitors who come to Bodrum for local food consumption [26].

Current research cannot be generalized as it has certain limitations. However, other studies carried out in the famous tourism destination, preferred as a research area, confirm that there are gaps in accessible tourism. In interviews with the managers of hotels accommodating disabled tourists in Bodrum, it was revealed that the hotels were not conscious of the accessible tourism market and that legal practices were insufficient [27].

## 3. Methods

### 3.1. Design

The qualitative research method, which is a research method that examines human and social behavior in social sciences, such as psychology, sociology and education, was used in the study based on the subject of tourism and disabilities [15]. In-depth interviews were conducted with semi-structured open-ended questions [15]. Before asking each participant the interview questions, they were sent information documents indicating the purpose and content of the research and a consent form to protect their personal rights. One year before the data collection date, four rehabilitation and health centers in Bodrum were visited, and a pilot study was conducted: preliminary interviews were held with PWDS and their families/caregivers to investigate their holiday intentions and the internal and environmental factors that helped them decide on a vacation, and questions were prepared in light of this preliminary study.

### 3.2. Data Collection

Separate semi-structured in-depth interviews were held with disabled individuals who had a holiday experience and used wheelchairs in the Bodrum destination. All interviews were conducted by the first author. A preliminary research project and a pilot study were applied to determine the main themes and questions to be asked. Participants were asked thirty-nine questions regarding their participation in travel and travel processes. Since the concept and diversity of disability have a wide scope, disabled individuals using wheelchairs were preferred in the study. Interviews were conducted after the ethical consent of the participants was obtained. It was thought that directing the questions to the participants at similar times would reflect positively in the study in terms of preventing them from being affected by seasonal changes. The questions were directed to the participants in Turkish, their own language, and then translated into English.

Thirty-nine open-ended questions were directed to 25 WCu online, audio recordings were transcribed and all the answers were prepared in the form of written documents. In light of the answers given, the keywords that emerged in the theoretical framework were defined and divided into titles according to the answers given to the questions.

As a result of the interviews conducted in the place and time specified in the scope, the descriptive and general evaluation analyses were reported and interpreted by separating the themes, and the determinants and keywords determined using the Nvivo program (QRS International, Melbourne, Australia) were coded.

### 3.3. Study Participants

WCu who were over 18 years old, had travel experience in the Bodrum region at least once, and these interviews, which they participated in voluntarily, were included in the study.

The first author contacted the Spinal Cord Paralytics Association of Turkey (SCPAT) and sent the information about the study to its members. When the participants met the inclusion criteria of the study, a consent form was sent to them and they were asked to participate voluntarily.

### 3.4. Limitations

Interviews were conducted online due to the COVID-19 pandemic. However, individuals with mental disabilities or hearing loss who could not answer the questions were not included in the study. Some of the participants also had chronic ailments, such as difficulty speaking, but they were included in the study because they were able to answer the questions asked.

## 4. Findings and Discussion

Interviews with each participant took an average of 30 min.

### 4.1. Samplings

A purposeful sampling technique was utilized in the research. The use of the sampling method is often preferred in qualitative research [28]. In this study, the reason why this was preferred is that WCu who have traveled to Bodrum for holiday purposes before and participated in tourism are experienced. In this way, we analyzed the tourism experiences of disabled participants to establish the determinants of their travel motivation and identify keywords of travel motivation. The demographic characteristics of the participants are given in Table 1.

According to the information in Table 1, the majority of the participants were men (*n* = 13). In addition, the remaining participants were female (*n* = 12). The ages of the participants varied. In terms of marital status, 20 (80%) participants were single, while five were married (20%).

According to participant age, two participants were between 18 and 25 (8%) years old, three participants were between the ages of 26 and 30 (12%), 16 participants were between the ages of 31 and 40 (64%) and four participants were between the ages of 41 and 50 (16%). In terms of education status, three participants (12%) were secondary school graduates, eight participants were high school graduates (32%), while more than half of participants (14; 56%) were university graduates. According to the monthly income levels of the participants, 17 participants’ (68%) income levels were under 1500 TL, three participants’ (12%) income levels were in the range of 3000–5000 TL, while five participants’ income levels were 20% over 5000 TL.

### 4.2. Disability Characterization of the Sample

Participants were mostly disabled individuals with spinal cord paralysis as a result of a traffic accident (13 people; 52%). Only one person had spinal cord paralysis as a result of a bullet injury (4%), and four people (16%) had multiple sclerosis. Two people (8%) had polio, and another two people (8%) had cerebral palsy. The other respondents (3 people; 12%) had spina bifida.

### 4.3. Determinants of Travel

**Figure 1:**
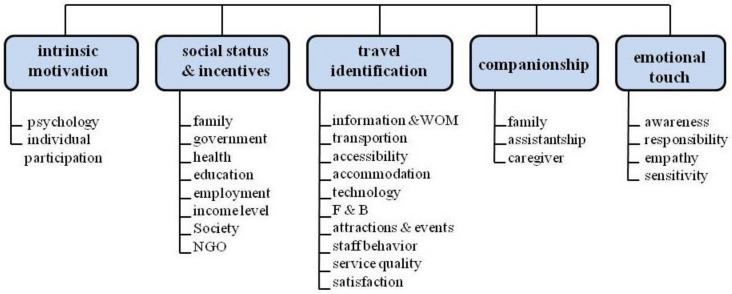
Determinants of travel for wheelchair users.

As a result of in-depth interviews with disabled individuals who used wheelchairs and had holiday experience, the factors and determinants affecting their travel decisions and travel processes were grouped and classified under headings with keywords in line with their answers to the questions (Figure 1).

- Intrinsic motivation: psychology and individual participation;
- Social status and incentives: family, government, health and education, employment, income level and society;
- Travel identification: process and result relationship;
- Companionship: family or assistant;
- Emotional touch: awareness, responsibility, empathy and sensitivity.

**Figure 2:**
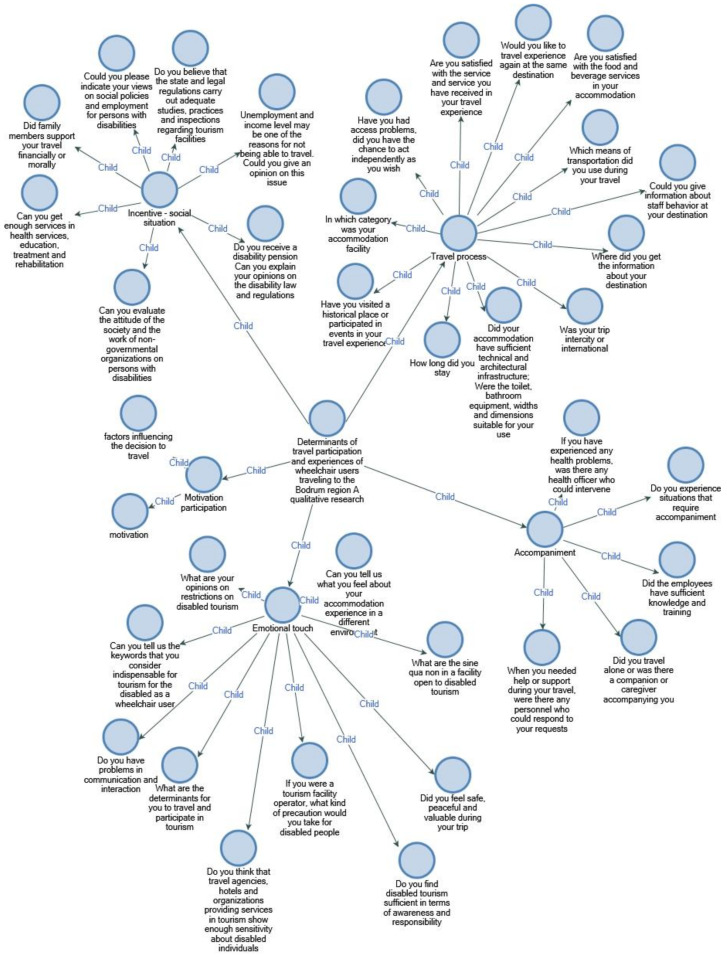
Determinants of travel participation and experiences of wheelchair users traveling.

Participants were asked 39 different questions in the research and were categorized in light of the answers given (Figure 2).

#### 4.3.1. Intrinsic Motivation and Participation

Individual participation is substantial for disabled individuals to participate in tourism activities. The person should be willing to travel and be psychologically ready to do so. Motivation and determination are required to be a tourist. An individual who thinks that travel criteria are available also tries to improve their traveling skills.

Many scholars mentioned with the multi-dimensional approach that the infrequent travel of PWDS is related to motivation and psychology, which emphasize accessibility concerns and restricted possibilities [29].

The determinants of the participation of PWDS in tourism and travel activities limited in their daily activities, the complex nature of their efforts to participate in tourism and the environmental factors that support or prevent participation have been examined by many researchers [30,31,32].

Motivation and participation were analyzed within the framework of the research. At this point, accessibility, taking on the tourist role and the intention of vacationing should be examined by the travel sector [33].

**Figure 3:**
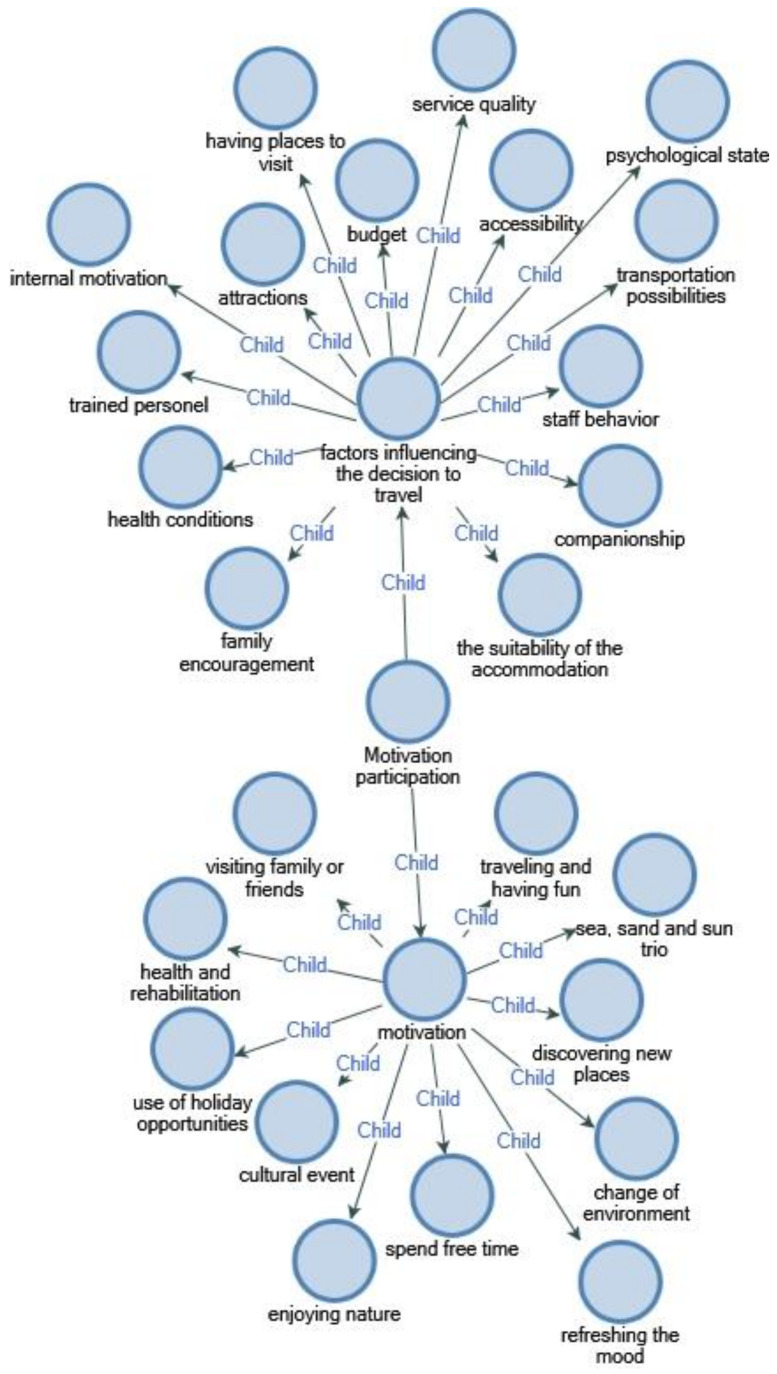
Motivation.

In fact, the results revealed that WCu want to travel for reasons such as exploring new places, having fun, changing their mood by changing the environment, and also that psychological state was the greatest motivation for participating in travel (Figure 3).

The results confirmed that the reasons for disabled people traveling vary, but the intrinsic motivation that influences their decision-making is affected by environmental factors. Stating that they intend to go on holiday provided that their needs of accommodation, transportation and access facilities are met, and the necessity of companions, disabled travelers stated that they can travel, especially if they have a sufficient budget.

The answer given by a WCu participant included in the study to one of the questions asked about travel participation is as follows.

“Like every human being, I always have the intention of participating in a trip where I can have activities such as swimming, sunbathing, visiting historical places, sightseeing and having fun, but in order to plan a vacation, you need a sufficient budget and most importantly, a strong psychological motivation to be ready for the difficulties you may encounter.”

#### 4.3.2. Social Status and Incentives:

- Family;
- Government and laws;
- Community and non-governmental organizations (NGOs);
- Health and rehabilitation facilities;
- Employment and income level;
- Education.

Disability, which emerged with the history of humanity, is a disadvantage that continues in today’s society. In different periods of history, different applications have been carried out for the disabled, and in the modern world, within the scope of the understanding of social state, the services for this group have diversified.

The support of family members, protection of their rights and freedoms through state and legal regulations, their involvement in education and employment, raising awareness of the society and assuming responsibilities and duties will facilitate the lives of disabled individuals and improve their social status.

Improving the social conditions of disabled people who have problems in accessing services that many people are accustomed to and can easily access will increase their quality of life, which will increase their participation in tourism and cultural activities. In fact, these results show that the budget and socio-economic status of disabled individuals play an active role in the decision to participate in tourism activities, as well as the elaboration of spatial and organizational services, such as transportation, access, convenience of accommodation and the provision of professional services tailored to individuals with special needs, enabling the realization of tourism for the disabled [34]. Apart from the inherent motivation and environmental impact of disabled tourists, tourism businesses may also need incentives to develop accessible tourism organizations and the disabled tourist market [35].

##### Family and Holiday

There is a wide network of support for PWDS to participate in tourism activities: family members and friends; resource agencies, which include the community, peer support groups, disability associations and professionals.

Little research has been conducted on the influence of family members on the participation of disabled people in travel and tourism. Participation in activities and reciprocal relations were examined in this study, which determined the effects of families on tourism motivation and the activities of individuals with disabilities. The permission and support of family members for the disabled family member to carry out tourism activities will facilitate the person’s participation in tourism [36].

##### Government

The recognition of rights and freedoms through state and legal regulations, and if it is necessary, the investigation of the suitability of tourism facilities for the use of persons with disabilities, can ensure that the availability of tourism facilities is increased through inspection, control, improvements and corrections.

The universal and fundamental freedoms of all humans in Turkey are indivisible, interrelated and interdependent with the nature of disabilities, which reaffirms the need to ensure the absence discrimination. This can be achieved through the utilization of the “Convention on the Rights of Persons Disabilities” United Nations Human Rights, assuming that everyone accepts and adheres to the rights and freedoms recognized by the Universal Declaration and the International Conventions of Human Rights and acts without any discrimination. These were decided by the Council of Ministers on 27 May 2009 in accordance with Law No. 244 [37].

Hence, the accessible tourism and hospitality sector is more than responding to the growing demand, and for an equitable society against discrimination, discriminatory approaches to customers with disabilities must be stopped. United with Turkey’s Constitutional Nations Rights UNCRPD research, which compares the agreement, the disability policy shows the compatibility of the constitution and bylaws but that Turkey should still improve the legislation concerning persons with disabilities, and reveals that the law needs to be changed [38].

Disability and access have been the subjects of numerous government regulations and coordination through disability law, human rights protection effort, awareness education and state-based tourism marketing authorities and policy participation [39,40]. The current research underlined the need for the harmonization of policies, recognition of democratic rights and freedoms, protection of values without discrimination and new regulations in order to maintain and fulfill the human rights of persons with disabilities [41].

##### Community and NGOs

PWDS are part of the society they live in. Reintegrating them into society and supporting them in overcoming the difficulties they encounter in daily life can only be possible if society and non-governmental organizations fulfill their duties and responsibilities. Karaatmaca and colleagues [42] conducted a content analysis to evaluate the service capacity of non-governmental organizations in disability services, and as a result, revealed that non-governmental organizations need awareness on disability.

The work of NGOs should not be limited to the commemoration of the International Day of Persons with Disabilities or occasional meetings, but the activities of disabled community organizations should be supported by financing, personnel, precautions and inspections of the movements of disabled persons [43].

##### Health and Rehabilitation Facilities

In order for a country to be mentioned among developed countries, the health service standards maintained within the ministry should be high, and there should be a treatment and rehabilitation system developed for the disabled [44]. The authors [45] aimed to fill a gap in the literature by investigating the obstacles to the administration of health and rehabilitation items in a study conducted on the representatives of disability organizations, NGOs and service providers. Health and rehabilitation are also defined as the human acts that allow people with disabilities and in need of care to engage in life, be relieved of future anxiety, use their leisure time productively and be sensitive and protective of their own body and living spaces. Nevertheless, the results of the responses of wheelchair users show that they generally do not benefit from health and rehabilitation services because they think that they are not likely to recover and walk again.

##### Employment

Many countries aim to develop and prioritize Active Labor Market Policies (ALMP) to help the unemployed and the disabled, preferably in normal employment to take part in business life. Human resource management, which is aimed to be a productive, cost-effective and flexible organization with a social policy strategy, will be able to provide employment solutions to disabled individuals in order to provide economic rationality, prosperity and social legitimacy [46,47].

The recent years, research has emphasized that the disability policy in European countries aims to increase the employment chances of disabled people by focusing on social investment; thus, it can be easier to overcome the wider barriers in society [48].

Developing an organizational climate of openness and tolerance, investing in centralized and good human resource management practices for disabled employees, managerial planning and operational processes are key elements for the integration of the disabled into the workplace (Gröschl 2013).

##### Education

Most disabled children cannot benefit from the right to education or do not have the necessary financial means to receive education. Disabled individuals are discriminated against in the field of education due to other students, teachers, parents and school administrators. Disabled individuals who cannot receive education have problems in not being able to participate in business life and integrate into social life.

Higher Education Institutions (HEIs) should partner with the government to include special education specialist teachers in schools for individuals classified as “disabled” to support inclusive and equitable education [49]. UNESCO, a private agency of the United Nations published a document with the United Nations Educational, Scientific and Cultural Organization in 2015; it underlined that the obstacles to education should be removed in order to ensure the full and equal participation of all disabled individuals in society.

The results of the study proved that social status, incentives and income level have a significant effect on the decision to go on vacation. Wheelchair users who could not complete their education as they wished due to disability and financial difficulties emphasized the importance of family incentives. The wheelchair users who were found to not benefit from health services, rehabilitation and treatment services sufficiently stated that they stopped the treatment because they were not likely to walk.

**Figure 4:**
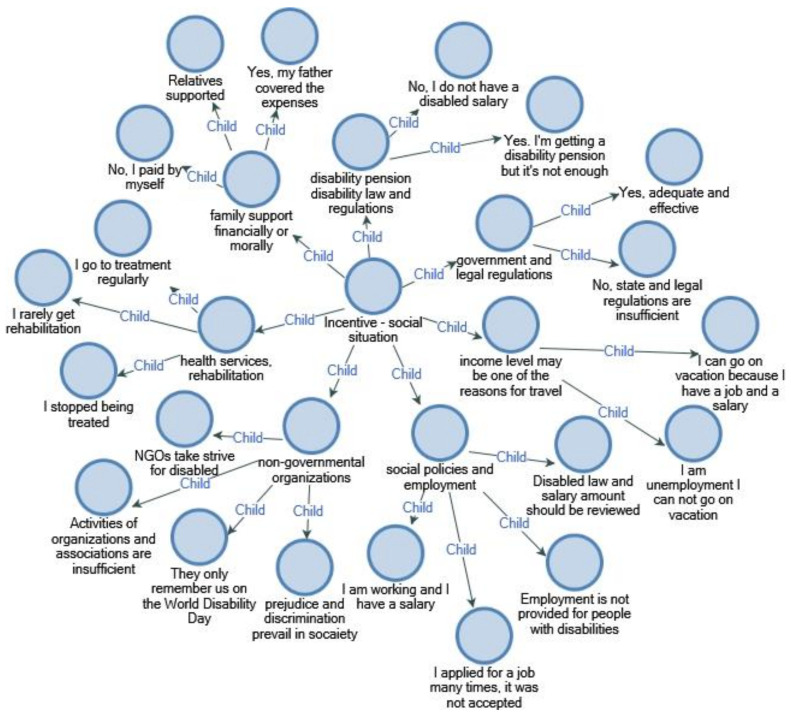
Social status and incentives.

As a result of the research, disabled people complaining about unemployment and low income stated that the state should review the issue of disability law and disability salary. The results of the research proved that civil society organizations are fighting for people with disabilities, but community support and participation are insufficient (Figure 4).

The answer given by a WCu participant included in the study to one of the questions asked about social status and incentives is as follows:

“I have applied for a job many times but they have not made a return, they do not include disabled individuals in business life, and employers generally think that we will not be productive, but there are even those who think that we will affect the psychology of other employees negatively. In addition, it is a luxury for me to take a vacation while the state’s disability pension is not even enough to meet my essential needs. There are laws and regulations regarding the disabled, but I think the extent of implementation is open to discussion.”

#### 4.3.3. Travel Identification

The travel organization of wheelchair users is divided into subheadings, namely, information retrieval, transportation, accommodation, F&B services, attractions, activities and visits to historical places, service quality and satisfaction.

- Information— word of mouth (WOM)—technology;
- Transport and access opportunities;
- The suitability of the accommodation and technological equipment;
- F&B;
- Visiting places, attractions and events;
- Staff behavior;
- Service quality and satisfaction.

Consequently, the travel organization of wheelchair users is divided into subheadings, namely, information retrieval, transportation, accommodation, F&B services, attractions, activities and visits to historical places, service quality and satisfaction. Many studies have been conducted to convey information about the infrastructure of the facilities and the services provided [50].

The disabled tourist travel and accommodation operation is a staged concept and needs to be detailed: The first step is to be willing to travel for any purpose and to make a decision; at this stage, information about the destination is collected through social media, internet or friend referral. Second, the means of transportation to reach the destination are investigated, and access opportunities are evaluated. In the third stage, the architectural suitability, comfort and technological infrastructure of the accommodation is examined. Fourth, details such as food and beverage services, restaurant or room service are investigated. At another stage, the possibility of participating in activities or actions suitable for the purpose of travel to the destination is evaluated. The sixth step is the communication and interaction of the staff with the customer, their attitude and behavior towards the customer and their training and knowledge, which are important details in the travel and tourism industry. Finally, the quality assessment of the products or services offered is among the most important stages that question customer satisfaction.

##### Information–Promotion–Booking–Technology

Although the number of people with disabilities is increasing, promotional tourism materials continue to maintain a homogeneous view that appeals to non-disabled people. In addition, physical and psychological factors that prevent disabled travelers from participating in travel are not adequately addressed [51].

Since the early 1980s, the effort to obtain information, communication and adapt to technological changes has created radical changes in the international tourism industry in developed and built up societies. Furthermore, searching for, obtaining information on, recognizing and even booking holidays in touristic places have integrated the concept of electronic tourism in the tourism industry at the international level, and by combining it with information and communication technology (ICT), the electronic identification of tourist sites has become a reality.

Emrouzeh and colleagues [52] focused on ICT that based disabled tourist and tourism action processes on four basic concepts: foresight, solution, quality assurance and product release; moreover, the investigation defined the relationship between the use of mobile applications, users and places in the tourism industry to achieve a better understanding of the following dimensions: navigation, social, mobile marketing, security, transactional, entertainment and information.

Technological developments and barrier-free information services for the disabled are constantly changing and updated solutions that have been discussed by researchers for disabled people to enter social life and communicate better with their environment, to make products and services suitable for the use of disabled people and to ensure that they benefit from high quality standards in all aspects of their lives. Social media, which is among the most used professions in our daily life, and marketing strategies in the tourism and hospitality sector, is the first choice of disabled travelers who want to obtain information while traveling—internet, navigation, applications, ICT, travel and tourism. New generation technology, innovation and inventions, developments and changes can make life easier for people with disabilities. Technological products that use artificial intelligence and the internet of things appeal to them as well. The number of inventions that affect their lives on the street, at work, in daily life, in treatment, education and even on vacation is increasing day by day. At this point, the tourism and travel industry should integrate developing technology and innovation into touristic facilities. [53,54,55,56,57]

Word of mouth (WOM), which enables a product, service or event to spread and reach large masses as a result of communication, has become a traditional marketing method used frequently on digital platforms and social media. It continues to exist in its most modern form today, as it consist of comments evaluating the satisfaction of a product or service as a result of the perception of the experienced professionals, and the success of reaching large masses in a short time [58]. Online comments have become an important tool for disabled guests to make decisions. Disabled guests are more likely to search internet comments for accommodation decisions than other guests. Electronic word of mouth (eWOM) comments are of great importance for disabled guests to evaluate a hotel’s responsiveness to expectations, such as personalized hotel service, location accessibility, hotel attitude and atmosphere, and to make decisions based on the comments made [59].

The results of the interviews show that many wheelchair users complain about information and technology use. There is a problem in obtaining information after the experience is compared with the preliminary information about the preferred tourist accommodation from agency, internet or friend recommendations. Technology and auxiliary products for the disabled can make daily life slightly easier. From mobility aids, such as walkers, wheelchairs, scooters, crutches and walking sticks, to medical devices and prosthetic limbs, new types of technology help cultivate the quality of life for disabled people.

##### Transportation

Transportation is among the most important stages of travel. Transportation problems in National Parks for disabled people have been researched, which mentioned that the population of disabled people, known as the minority community, is increasing day by day, and the problems experienced by disabled tourists are increasing in parallel [60]. Soltani and colleagues [61] emphasized that PWDS experience more difficulties and access problems while traveling and using public transportation.

Another investigation concerned the flight experiences of disabled individuals using wheelchairs; the emotions they experienced during the transportation phase were included; access difficulties and crew behaviors were discussed; physical and social difficulties determining whether they needed a companion were expressed; and air transport and studies on tourism management were highlighted [62].

In a study on the problems with the regulation of disabled transport, it was revealed that a new generation bus with a ramp designed to transport disabled people in wheelchairs is rarely seen in any city [63]. Despite the rapidly growing elderly travel market and the number of disabled tourists, the tourism industry has paid little attention to these tourists’ characteristic choices for accessible travel products.

##### Suitability of Accommodation

The act of going to a place other than the home where the disabled person lives and finding accommodation is possible only if the internal and environmental conditions are fulfilled. Among the most important determinants of the holiday, the destination and the suitability of the accommodation are issues that should be examined in depth for disabled travelers [64].

In order for the disabled individual to perform a tourism activity, there must be a facility that meets their needs and special requirements, such as the suitability of the accommodation, the adequacy of the technical equipment, the architectural structure, the knowledgeable staff or the accompaniment of a companion.

The results show that the travels of wheelchair users take an average of 3–4 days, and generally mid-level hotels, such as boutique hotels, apart hotels or 4-star hotels, are preferred.

While well-financial travelers prefer 5-star hotels and have an average of 5 days of vacation, it is observed that disabled people with low income and employment problems prefer to stay in small-scale hotels.

Disabled tourists, who generally made negative comments about the technical infrastructure and architectural structure of the hotels, underlined the frequency of access problems in touristic facilities.

##### F&B Services

Nutrition and disability are interrelated. UNICEF is among the most widespread and recognized social aid organizations in the world, providing humanitarian and developmental support to children, and recommending the rejection of discrimination in matters such as food and beverage and health services.

Disabled people may have difficulty in consuming the appropriate type and sufficient amount of food orally, which leads to malnutrition. In disabled children, problems such as refusing to eat, choosing a meal, vomiting after eating, undereating, inability to swallow, pushing with the tongue, keeping food in the mouth for a long time are observed. The nutrition of individuals with special needs is of paramount importance, because the foundations of lifelong health, strength and intellectual ability are laid in childhood.

Health tourism is a type of tourism that gathers different segments, such as accessible tourism, medical tourism and spa and wellness tourism, which are increasingly important in the world. Food and beverage services for tourists participating in health tourism differ from the food and beverage service offered to normal tourists in terms of quality, variety, hygiene and presentation styles. The expectations for each sub-segment of health tourism and F&B services were examined [65].

The relationship between the problems encountered by disabled individuals in catering establishments, the problems faced by the participants and the frequency of visits were examined [66]. Hence, to improve the nutrition and education of students with special needs, regular nutrition policy planning and awareness programs for teachers, resources, trainers, experts and parents are needed.

##### Attraction–Entertainment–Events

While physical activity plays a significant role in psychological, physiological and social concepts for all people, it is more important for disabled people. In addition, the state should take roles in directing disabled individuals to physical activity with future facilities, organizations and sponsorships. In addition, media, educational institutions and public institutions should play a role in raising the awareness of the whole society, especially families with disabilities, about physical activity for the disabled [67].

PWDS are deprived of many activities, such as visiting museums or historical places, excursions and tours, festivals, sports, concerts, plays and competitions. It is necessary to maintain the full and effective participation of persons with disabilities in social life and activities under equal conditions with other individuals by encouraging and ensuring the benefits of fundamental rights and freedoms and by strengthening the respect for their innate dignity, and to ensure that necessary arrangements are made to take measures to prevent disability.

The reasons why individuals with disabilities do not participate in activities are investigated in a previous paper [68]. The reasons for the disabled individuals not to participate in the activities are examined under two main headings: individual and environmental factors—psychological, economic, social, family, gender, companion needs, community participation and encouragement, facilities and materials, clubs and associations [68].

Another study in Finland, where national programs should be implemented to encourage physical activity, measured the average daily movement distance and duration of wheelchair users and collected user experiences by examining the effect of an activity tracker and mobile application on their movement levels [69].

##### Staff Behavior

Millions of disabled people cannot benefit from vacation opportunities, despite having financial means, due to the obstacles caused by the attitudes of the managers in hotels, the lack of education or the behavior of the employees.

Although the regulations have been mandatory in the last 10 years since the Barrier-Free Accessible Architectural Environment Law and the Barrier-Free Transport Law enacted in 2005, no solution has been provided, except for a few steps for show. There are hardly any facilities where disabled people, elderly people and their families can have a holiday together with other people. Of course, making architectural arrangements is not enough for the participation of disabled people and their families in life. In order to change mentalities, public officials, private sector employees and the whole society should be given training on understanding the disabled, communicating without marginalizing and correct behavior.

Hospitality and tourism management should pay attention to the issue of communication and interaction with the disabled within the practices of service delivery: prejudice or hidden attitudes, unconscious movements in contact with the disabled person or negative behavior will harm accessible tourism and negatively affect the social structure of the tourism sector [70]. In addition to transportation and access issues in the accessible tourism industry, disability-friendly staff behavior is also of great importance to ensure tourist satisfaction [71].

An investigation of the problems indicated the experiences of disabled individuals while receiving tourism services from their posts on complaint websites and examined the issues of complaint. The results of the research revealed that the issues most frequently complained about by the disabled individuals are employees’ negative attitudes and behaviors towards disabled tourists [72].

##### Service Quality and Satisfaction

The most disadvantaged group in hotels was physically disabled people, those using electric chairs and wheelchairs, which was the result of the study investigating the hotel satisfaction of individuals with physical disabilities who need to use assistive devices and have access problems [73].

The results show that disabled travelers generally prefer internet and social media tools for information, reservations and making short-term trips. WCu staying in 4–5 star hotels said that they would like to stay in the same hotel again, while those who stay in boutique and apart hotels stated that they want to go to the same destination but do not want to stay in the same hotel.

Stating that they are mostly dissatisfied with the technical and architectural structure of the hotel, the participants underlined that wheelchair users experience access problems.

According to the results, it is observed that participation in activities, such as events and historical site visits, is low.

In addition, the participants stated that there is mostly a moderate level of satisfaction with regard to the questions about food and beverage services, service quality and satisfaction, and stated that the services offered by hotels for disabled tourists should be reviewed.

**Figure 5:**
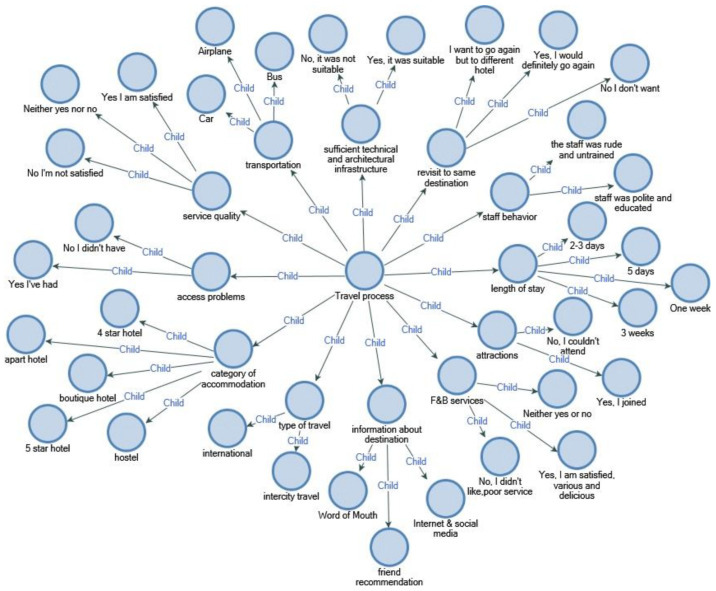
Travel process.

The participants emphasized that staff are mostly unequipped and uneducated, and that experts who understand health and psychology should definitely work as hotel staff. When the participants were asked whether they would like to travel to the same place again, they stated that they generally wanted to travel to Bodrum city again, but they did not want to stay in the same hotel (Figure 5).

The answer given by a WCu participant included in the study to one of the questions asked about travel identification is as follows:

“My travels start with great excitement and end with disappointment. I think that hotels are not prepared and supervised enough to serve individuals with wheelchair users. In the last hotel I stayed, we removed the door of the bathroom because my wheelchair could not fit, and we used this door as a ramp because I had an access problem at the entrance of the room. In addition, the hotel did not offer the services it exaggerated during the information and reservation phase, for example hotel staff were mostly part-time students I once felt a lot of pain in my body due to the wrong intervention of an employee who attempted to help me and I also ordered food in the room because I had chronic illnesses, swallowing and speaking difficulties, but I did not get the service I expected.”

#### 4.3.4. Accompaniment–Assistantship

Most of the individuals using wheelchairs also have other types of disabilities and ailments and even have to receive treatment and health services at certain time intervals, so these individuals need to seek professional help many times in their daily life. Considering the independent movements of the disabled person inside or outside tourism facilities, the need for a companion or assistant comes to the fore. Among the rational solutions of tourism for the disabled will be to include a professional who has received training, such as physiotherapy, health and rehabilitation, in the staff; this will provide opportunities in the tourism and hotel sector for healthcare providers in terms of employment, and increase the satisfaction of the individual in tourism activities by raising the quality standards of the service provided to the disabled person.

Disabled people’s activities and travel patterns are more dependent on others due to their special physical conditions, meaning that the vacation needs of physically disabled travelers depend mostly on accessibility, and at this point, they often need a caregiver, assistant or companion [74].

Undoubtedly, when the accessibility of accommodation facilities is ensured and the diversity of activities reaches standards suitable for the physically disabled, the opportunities for disabled travelers to use the facility will increase. For this reason, disabled people and their families want to choose the facilities where they can have a holiday just by observing. Improving the ergonomics of the rooms of accommodation facilities so that physically disabled individuals do not need the help of their families psychologically puts the disabled and their families into a holiday atmosphere. The ergonomic conditions in hotels are the general standard measures specified in the regulations and, therefore, may not be suitable for the expectations of disabled tourists [11]. At this point, employing specialist physiotherapists, healthcare professionals or special education teachers in tourism facilities will ensure that the disabled tourists have holidays with the least problems. Many researchers examined the tourism activities of disabled people with their caregivers or accompanying caregivers [75]

Physically disabled individuals and wheelchair users need professional help and special care often in daily life due to their inability to provide their own care. Wheelchair users need an assistant or caregiver in order to meet their needs, such as bathing and showering assistance, changing clothes, meal planning and medication reminders. The previous studies were examined, it was revealed that among the biggest gaps was the need for residents of PWDS called attendants or caregivers. When the results of numerous interviews and studies conducted with disabled individuals are examined, it is seen that generally family members assume private care duties. It is possible for individuals with financial means to receive special care, health and rehabilitation services. In order to recognize equal rights and freedoms and to meet the needs of disabled people, the tourism industry needs personnel who can provide special service support to people with disabilities.

The results show that WCu find it almost impossible to travel alone while meeting their daily needs; therefore, the support of a companion or caregiver is among the expectations.

The researchers [76] emphasized that the family members or caregivers accompanying disabled people often have difficulty traveling alone due to various restrictions and obstacles, and the satisfaction of caregivers accompanying the disabled tourist is a special need, and underlined that this increases the quality of the service environment.

Additionally, travel agencies are an important resource for disabled people to purchase travel products; the information provided by travel agencies has a great effect on successful travel or an unsatisfied trip [77].

The disabled tourist market is evolving globally, but tour guides and their contribution to the tourist experience are given sufficient attention. The interaction between the tourist and the tour guide is important in determining the types of holiday and meeting the demands in the travel process [78].

**Figure 6:**
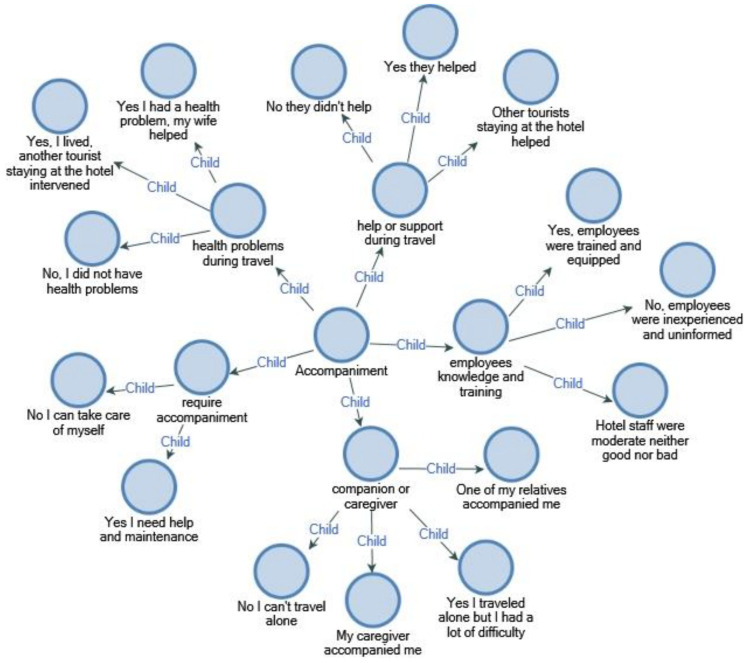
Accompaniment.

The answer given by a WCu participant included in the study to one of the questions asked about companions is as follows (Figure 6).

“I think that the only condition for having an enjoyable holiday that I will be pleased with as a wheelchair user is the companion issue. When I needed something during my holidays with my family members, they came to my aid and tried to fulfill my requests but I had a hard time when I was on vacation alone. After all, in the name of responsiveness, hotels are blocked at some point and I believe that this problem may be solved if healthcare professionals or special education specialists are included in the hotel staff. This both provides employment for them and strengthens the possibility of people with disabilities to stay in tourism facilities. There are many people with disabilities who do not go on vacation because they cannot pay for their private nursing staff or because family members cannot accompany the travel.”

#### 4.3.5. Emotional Touch

Disabled individuals who are exposed to negative behaviors, such as marginalization, discrimination, disrespect and exclusion, have problems with communication and interaction.

People with disabilities are confronted with an existing disability or health problem many times every day; their movements and actions are limited, so they require support, understanding, sensitivity and responsibility because of their high psychological sensitivity.

When mental and ethical barriers are added to their physical disabilities, living conditions become increasingly difficult for disabled individuals. For example, many families express denial, inability to accept, disappointment, guilt, fear, anger, despair, etc. These processes negatively affect the psychological adjustment process of the disabled member of the family.

We must adopt a sensitive attitude to try to understand them through empathy, to respect and protect their rights and freedoms and to do our part to make life easier for them.

Every individual has the possibility of injury, physical or mental discomfort, loss of movement, senses and functions in life, so everyone is a candidate for disability. With approaches such as establishing positive communication, helping, giving priority, using the right style, seeing them as fulfilling responsibilities rather than performing duties, we can enable them to improve their freedom and independence conditions.

Tourism sector employees and service providers should demonstrate a sensitive and stable attitude to provide opportunities that will provide peaceful, hopeful and safe environments to disabled individuals, who are an integral part of society.

Dovidio and colleagues [79] emphasized that studies on subjects such as antipathy towards physically disabled people, contemporary attitudes, social psychology and prejudice and discriminatory behavior towards these individuals are insufficient.

**Figure 7:**
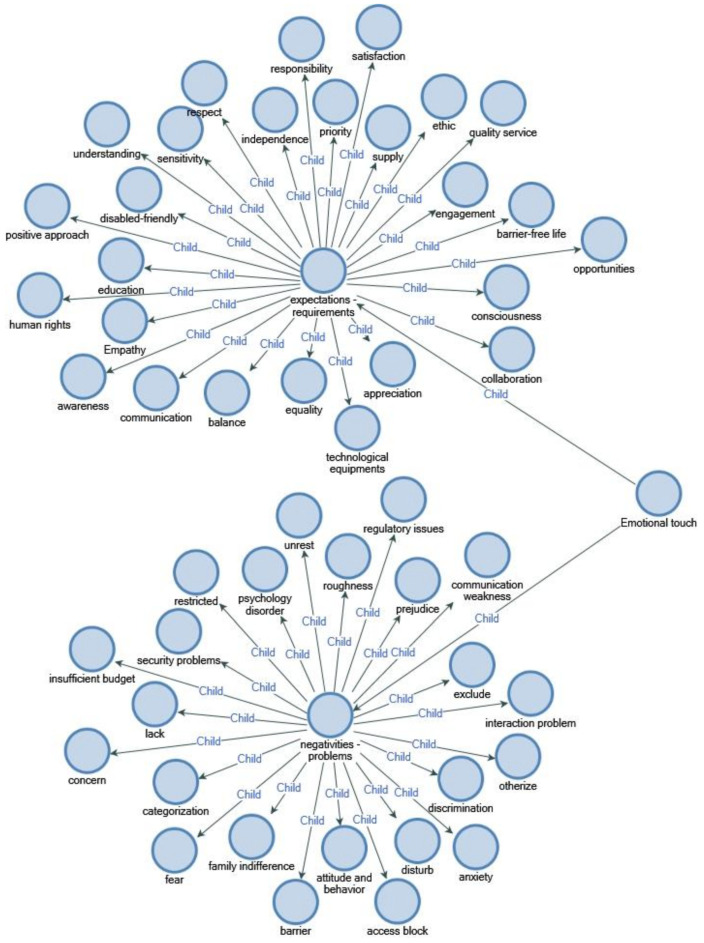
Emotional touch.

The answer given by a WCu participant included in the study to one of the questions asked about emotional touch is as follows (Figure 7).

“In my opinion, society is insensitive and unconscious about disabled individuals. They usually approach us with pity and we are marginalized. Because of discrimination and prejudice, many disabled individuals are afraid to go out, let alone go on vacation. I hope that he will adopt a more mindful approach, considering the possibility that one day everyone may experience obstacles. The barriers we face huge barriers not only in access, but also in minds, attitudes and behavior. We don’t demand anything impossible. We want them to touch our hearts and respect us with sensitive touches that include responsibility and awareness. In order for us to participate in tourism or other activities and to enjoy rights and freedoms equally as other people, abstract concepts should be given more importance.”

##### What Is a Wheelchair?

A wheelchair which is a medical chair used when walking is difficult or impossible due to a physical discomfort or injury. Wheelchairs can be manufactured individually for the user, as well as having variations that allow the person to be pushed manually by providing manual control. There are handles on the back of the seat that allow different individuals to push it.

Different wheelchairs have different features and different prices. When choosing a wheelchair, you must first choose the suitability of your patient, that is, you need to determine which chair is suitable for your patient, and then it is necessary to determine the appropriate products for all these conditions in the budget and medical stores you can reserve for the chair. A wheelchair may be within your budget, but if it is not suitable for the patient, it may be left unused (Table 2).

##### Types of Wheelchair and Tourism

The revealed that the type of wheelchair they use for disabled travelers is of great importance in terms of tourism activities. People who travel mostly to sunbathe on the beach or swim in the sea stated that the manual wheelchairs they use in daily life are not suitable for use on the sand (Table 2).

##### Word Cloud

**Figure 8:**
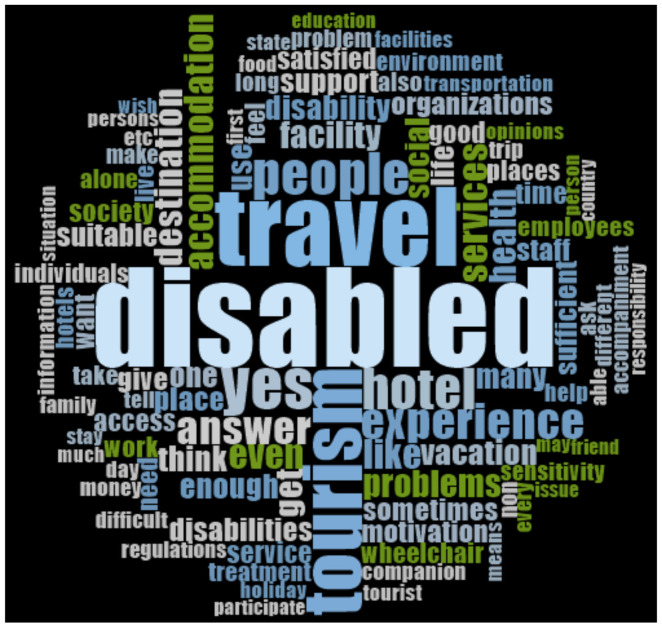
Computer software NVivo program was used for structuring the qualitative research conducted, analyzing the findings in an organized way and detailing frequently used keywords under titles.

The computer software NVivo program was used for structuring the qualitative research conducted, analyzing the findings in an organized way and detailing frequently used keywords under titles (Figure 8).

## 5. Discussion

The information in the current studies indicates the importance of intrinsic motivation for PWDS to go on vacation. Furthermore, it is a motivation source for the decision to participate in the holiday in social conditions, such as family incentives and budget status. The factors that affect the disabled individual’s decision to go on vacation and the determinants of this process are related to each other like a chain link.

Overall, WCu have high holiday intentions and tendency to travel; the number of days they spend on vacation during the year is very low. This may be caused by the possibilities that may occur at the holiday destination. Many disabled tourists argued that the promised service and the experience did not coincide with each other; therefore, they were misinformed. Access to information is important when traveling with disabilities.

As Domínguez Vila, Alén González and Darcy (2019) [80] point out, knowledge is not only a communication and marketing channel for tourism but also an experience transmitter. At this point, reliable comments can be accessed through word of mouth and e-complaints, and the opportunity to review comments about the travel experience can help users to obtain accurate information and make decisions [59,81,82].

Consequently, disabled individuals, many of whom are unmarried or divorced, emphasized the importance of family members’ incentives and support for travel, while underlining that the state’s legal regulations do not take adequate measures for accessible tourism. It is controversial to expect disabled individuals who are not employed, have low income levels and complain that vacation becomes an extra luxury issue for them because it is a costly action to participate in tourism activities.

The results of the interviews show that many wheelchair users complain about the use of information and technology. There is a problem in obtaining information after comparing the prior knowledge and experience of the preferred tourist accommodation with agency, internet or friend recommendations.

Although there are many models and scales related to service quality measurements and satisfaction assessments in the literature, it is revealed that these scales should be developed when the disability status and special needs of disabled individuals are examined. Someone else’s care, assistance or support—companions or assistants—is mandatory to meet the special needs of people with disabilities

However, another subject of discussion is the role and importance of wheelchair types in the life of the disabled individual. As a result of the in-depth interviews conducted, it was observed that the use of manual and electric wheelchairs was common. However, disabled individuals who go on vacation to experience the trio of sea, sand and sun experience problems of swimming from the beaches and mobility in the sand.

Types of wheelchair are increasing day by day with the developing industry and technology integration. PWDS who spend most of their time at home, at work, in treatment or outside generally prefer manual or electric wheelchairs that are compatible with their environment. Facilities open to tourism for the disabled during the summer months should review the issue of technical vehicles and equipment, and even provide wheelchairs suitable for beach use if necessary.

In general, it is observed that the participation of disabled individuals with low income and unemployment in trips, travel and tourism is insufficient. Due to the architectural obstacles in hotels and the barriers caused by the lack of education of managers and employees, millions of PWDS cannot benefit from holiday opportunities, although they have financial means.

The abstract concepts that we examined under the title of sensitive touch are the subjects that most affect the travel and daily lives of PWDS using wheelchairs. Tourists with disabilities complaining about the lack of education of the staff of facilities providing travel and tourism services revealed the deficiencies in social awareness and education. Finally, this article provides preliminary data for WCu in accessible tourism and shows results in the literature on their travelling experiences, limitations which they encounter when traveling under the name of accessible tourism and how these limitations affect their travel experiences. Accordingly, we think that our findings will guide various occupational groups related to disability and tourism.

## Figures and Tables

**Table 1:** Socio-demographic profile of the sample.

Participants	Gender	Marital Status	Age	Education	Income Level
**1.**	Male	Single	24	High school	Under 1500 TL
**2.**	Male	Single	28	High school	Under 1500 TL
**3.**	Male	Single	32	University	3000–5000 TL
**4.**	Male	Single	30	University	Under 1500 TL
**5.**	Male	Single	37	High school	Under 1500 TL
**6.**	Male	Single	33	High school	Under 1500 TL
**7.**	Male	Single	36	University	Over 5000 TL
**8.**	Male	Single	31	University	Under 1500 TL
**9.**	Male	Single	36	High school	Under 1500 TL
**10.**	Male	Single	50	University	Over 5000 TL
**11.**	Male	Single	41	Secondary school	Under 1500 TL
**12.**	Male	Single	31	University	Under 1500 TL
**13.**	Male	Single	36	University	Under 1500 TL
**14.**	Female	Single	38	University	Over 5000 TL
**15.**	Female	Single	35	High school	Under 1500 TL
**16.**	Female	Single	39	University	Over 5000 TL
**17.**	Female	Single	47	University	Under 1500 TL
**18.**	Female	Single	27	University	3000–5000 TL
**19.**	Female	Single	37	Secondary school	Under 1500 TL
**20.**	Female	Single	36	High school	Under 1500 TL
**21.**	Female	Married	25	University	3000–5000 TL
**22.**	Female	Married	38	High school	Under 1500 TL
**23.**	Female	Married	38	Secondary school	Under 1500 TL
**24.**	Female	Married	36	University	Under 1500 TL
**25.**	Female	Married	45	University	Over 5000 TL

TL = Turkish Lira.

**Table 2:** Types of wheelchairs reviewed in the published literature.

Manual Wheelchairs	Flemmer, C.L., & Flemmer, R.C.	2016
Electric Wheelchairs	Jones, M.A., McEwen, I.R., & Neas, B.R.	2012
Sports Wheelchairs	Cooper, R.A., & De Luigi, A.J.	2014
Powerchair Football—Power Soccer Wheelchairs	Richard, R., Perera, E., & Le Roux, N. Jeffress & Brown	2019 2017
Standing Wheelchairs	Shields, R.K., & Dudley-Javoroski, S.	2005
Pediatric Wheelchairs	Rispin, K., & Wee, J.	2014
Beach Wheelchairs or All Terrain Wheelchairs	Volk, M., Alderman, H., Blumenthal, K.R., Cooperman, B., Henry, D., Herman, L.A.,… & Granger, R.W.	2005

## Abbreviations

ALMP	Active Labor Market Policies
F&B	Food and Beverage
eWOM	The electronic word of mouth
HEIs	Higher Education Institutions
ICT	Information and Communication Technology
NGOs	Non-Governmental Organisations
PWDS	People With Disabilities
TOFD	Spinal Cord Paralytics Association of Turkey
TSI	Turkey Statistical Institute
TÜRSAB	Türkiye Seyahat Acentaları Birliği (Turkey Travel Agencies Association)
UNESCO	the United Nations Educational, Scientific and Cultural Organization
UNICEF	the United Nations Children’s Fund
WCu	WheelChair Users
WHO	the World Health Organization
WOM	Word of Mouth
